# Novel indoor positioning system based on ultra-wide bandwidth

**DOI:** 10.1186/s42492-019-0038-1

**Published:** 2020-01-07

**Authors:** Zhen Wei, Rui Jiang, Xing Wei, Yun-An Cheng, Lei Cheng, Cai Wang

**Affiliations:** 1 School of Computer and Information, Hefei University of Technology, Hefei, 230009 China; 2 Engineering Research Center of Safety-critical Industry Measure and Control Technology of Ministry of Education, Hefei, 230009 China; 3 School of Mechanical Engineering, Hefei University of Technology, Hefei, 230009 China

**Keywords:** Indoor positioning, Ultra-wide bandwidth, Position algorithm, Hardware platform

## Abstract

To tackle challenges such as interference and poor accuracy of indoor positioning systems, a novel scheme based on ultra-wide bandwidth (UWB) technology is proposed. First, we illustrate a distance measuring method between two UWB devices. Then, a Taylor series expansion algorithm is developed to detect coordinates of the mobile node using the location of anchor nodes and the distance between them. Simulation results show that the observation error under our strategy is within 15 cm, which is superior to existing algorithms. The final experimental data in the hardware system mainly composed of STM32 and DW1000 also confirms the performance of the proposed scheme.

## Introduction

The development of wireless communication technology has a deep impact on everyday life, and further attention is now being paid to service-based positioning [1]. Today, GPS is suitable for outdoor positioning; however, the indoor environment is complex, and it is more difficult to obtain accurate GPS information in this setting. There are many indoor wireless communication methods used, each with their own advantages and disadvantages. These include Wi-Fi, Bluetooth, infrared, ultrasonic, and radio frequency identification [2].

Although indoor positioning technology is in the early stages of development, it has nevertheless attracted the attention of many researchers. Excellent indoor positioning systems require high-precision positioning and low cost for a wide range of applications. As ultra-wide bandwidth (UWB) has both these advantages, this research proposes an indoor positioning system based on this technology. Hence, the proposed scheme adopts an improved positioning algorithm, with low complexity and high precision.

UWB as a strong ability to resist multipath interference [3], so indoor distance measurement can be achieved with high precision. UWB wireless communications research has been conducted at Stanford University, Berkeley University, MrrLincoln Laboratory at Massachusetts Institute of Technology, Mitsubishi Research Institute at the University of Southern California and Intel Wireless Research. Among them, Professor Robert A. Schultz is one of the key proponents of UWB technology development.

There are many positioning algorithms based on distance, including the Fang algorithm, Chan algorithm, and the Taylor series expansion algorithm. As the first two algorithms cannot eliminate the error that is caused by the multipath effect, the positioning accuracy is low. Therefore, this paper concentrates on the Taylor series expansion algorithm, as this is considered the best candidate for effectively processing distance information.

## Methods

### A brief introduction of UWB

UWB technology differs from traditional communication technology, in that it uses a nanosecond narrow pulse, which is non-sinusoidal wave, to transmit data and sends signals with extremely low power over a wide spectrum. In 2002, the Federal Communications Commission (FCC) defined UWB as follows: The relative bandwidth of the signal is greater than 20% or the absolute bandwidth is greater than 50 MHz. This conforms to the restriction of FCC of the power spectral density. The formula is shown as follows:

1 $$ {F}_B=\frac{f_U-{f}_L}{f_m} $$

**Fig. 1:**
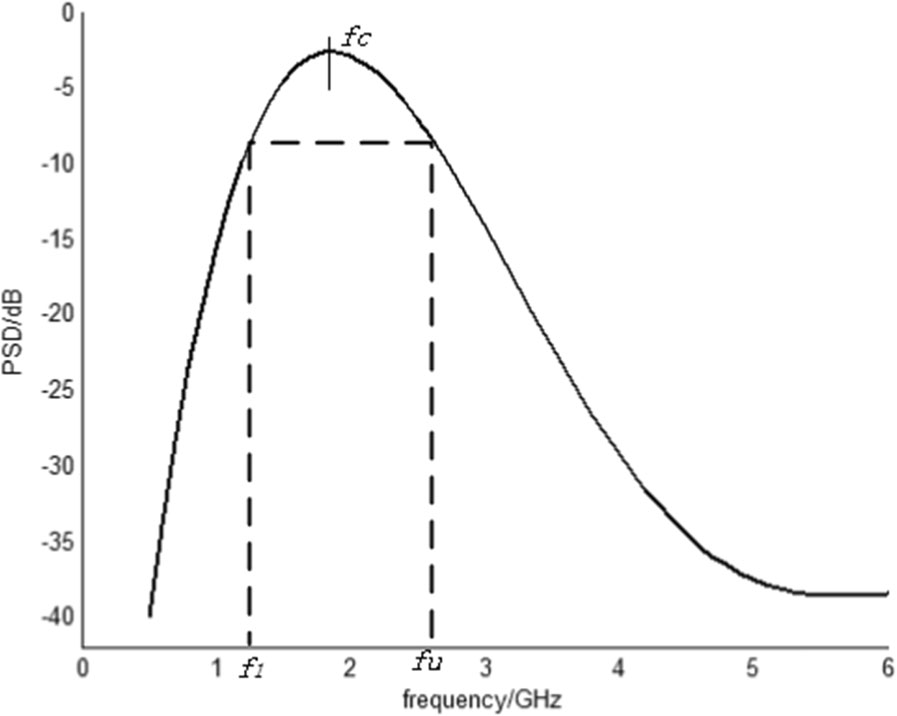
Definition diagram of ultra-wide bandwidth

In the formula, *F*_*B*_ represents the relative bandwidth; *f*_*U*_ represents the frequency of the highest point in which the antenna transmits − 10 dB radiation; *f*_*L*_ represents the frequency of the lowest point in which the antenna also transmits − 10 dB radiation; and *f*_*m*_ represents the center frequency, *f*_*m*_ = (*f*_*U*_ + *f*_*L*_)/2. Figure 1 shows the definition diagram of UWB.

The FCC document introducing the parameters of UWB only defines bandwidth value in principle; however, it does not set a limit on how to generate the UWB signal. In practical applications, to generate UWB signals that meet the specified parameters, impulse radio and multi-band are typically used.

### Characteristics of UWB

The main characteristics of UWB technology are as follows:

1. Low cost and low power consumption

The RF front-end, analog and digital signal processing components of the UWB system are relatively simple; hence, it is more straightforward to realize the full digital structure at low cost. The FCC strictly limits the level of transmission power in UWB. In short distance communication systems, the power of a UWB transmitter is typically lower than 1 mV. The system functions normally with a very low power supply, so it is a highly appropriate choice for battery operated equipment.

2) High data transmission rate with significant system capacity

2 $$ \mathrm{C}=\mathrm{Bl}{\mathrm{og}}_2\left(1+\mathrm{S}/\mathrm{N}\right) $$

According to the Shannon formula above, B represents the wideband of channels, and S and N represent the average power of signal and noise respectively. S/N represents the signal-to-noise ratio. Under the condition of certain S/N, the maximum information transmission rate of the system is proportional to the bandwidth. Because the UWB system uses a gigabit frequency band, the system can achieve a high transmission rate [4].

3) Strong penetrating force

As the UWB signal contains many low frequency components, it has strong penetration ability. The UWB can penetrate indoor obstacles like walls and floors. Therefore, it is highly suitable for indoor positioning with high precision.

4) Strong anti-interference ability

UWB scatters weak radio pulses across a very wide frequency range, and this effectively avoids interference with other systems. The duty ratio of the UWB signal is very low and uses the window function to eliminate signal interference; this enhances the anti-interference ability and security of the system.

### The distance measuring principle of UWB

UWB uses a double-sided two-way ranging method to measure distance between two devices.

**Fig. 2:**
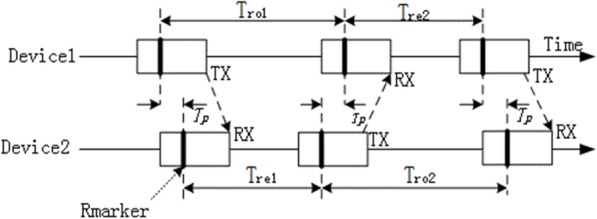
Schematic diagram of time difference of arrival algorithm

As Fig. 2 illustrates, the process of distance measurement contains six steps:

1. Device 1 sends the poll package and records the sending time T1;
2. Device 2 receives the poll package and records the time T2;
3. Device 2 sends the response packet at time T3;
4. Device 1 receives the response packet and records the time T4;
5. Device 1 sends the final packet and records the time T5;
6. Device 2 receives the final packet and records the time T6.

We calculate the time using four formulas:

3 $$ \mathrm{Tro}1=\mathrm{T}4-\mathrm{T}1 $$

4 $$ \mathrm{Tro}2=\mathrm{T}6-\mathrm{T}3 $$

5 $$ \mathrm{Tre}1=\mathrm{T}3-\mathrm{T}2 $$

6 $$ \mathrm{Tre}2=\mathrm{T}5-\mathrm{T}4 $$

The time required for the UWB signal flight is calculated using the following formula:

7 $$ \mathrm{Tprop}=\frac{\left(\mathrm{Tro}1\times \mathrm{Tro}2-\mathrm{Tre}1\times \mathrm{Tre}2\right)}{\left(\mathrm{Tro}1+\mathrm{Tro}2+\mathrm{Tre}1+\mathrm{Tre}2\right)} $$

The distance is calculated by Tprop multiplied by the speed of light.

## Results and discussion

### The principle of time difference of arrival algorithm

There are multiple methods used for indoor positioning, such as Received Signal Strength Indication (RSSI), Angle of Arrival (AOA), Time of Arrival (TOA), and Time Difference of Arrival (TDOA) [5]. Because TOA and TDOA positioning algorithms estimate distance based on the time of signal propagation, they can make full use of the advantages of UWB. Therefore, they are considered more beneficial than RSSI and AOA. However, the TOA position algorithm requires precise time synchronization between the two devices, which is difficult to achieve in hardware. Thus, in this study, we adopt the TDOA algorithm as the most appropriate distance algorithm to be used for the purposes of this research.

**Fig. 3:**
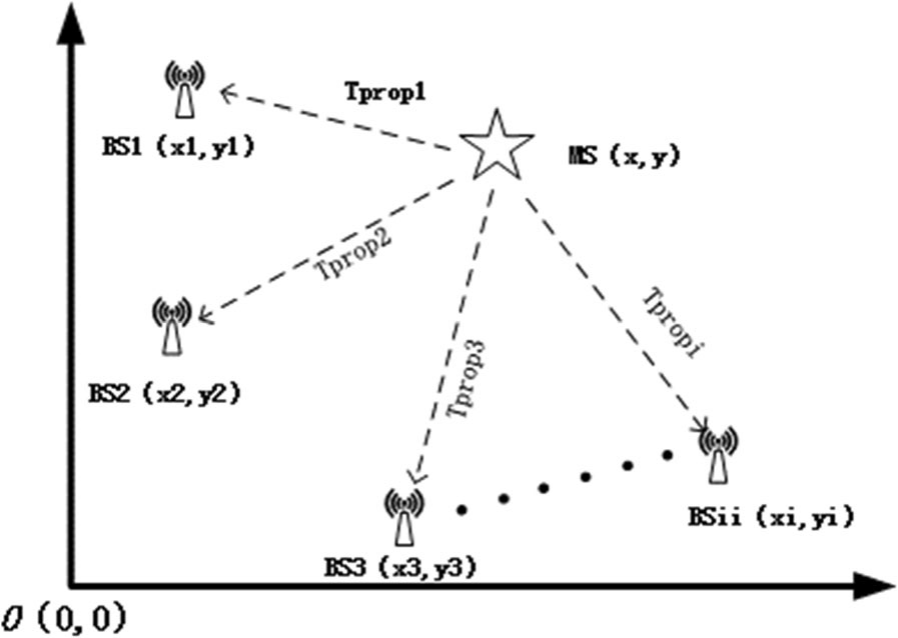
Schematic diagram of positioning principle

As shown in Fig. 3, BS_i_ (x_*i*_, y_*i*_) represents the coordinates of the base station, and MS (x, y) represents the coordinates of the mobile station, the Tporpi represents the time taken for the signal to transmit from the mobile station to the base station BSi, which is calculated in section 2.3.

The distance between the mobile station and each base station is calculated as follows:

8 $$ {\mathrm{S}}_i=\sqrt{{\left(\mathrm{x}-{\mathrm{x}}_i\right)}^2+{\left(\mathrm{y}-{\mathrm{y}}_i\right)}^2} = c\times \mathrm{Tporpi} $$

In this formula, *c* represents the speed of light, and S_*i*_ represents the distance from the mobile station to the base station *i*.

BS_1_ is set as a reference station. Formula (8) minus Formula (8) whose *i* = 1, then we can get position equation.

9 $$ {\displaystyle \begin{array}{c}{\mathrm{S}}_{i1}=\sqrt{{\left(\mathrm{x}-{\mathrm{x}}_i\right)}^2+{\left(\mathrm{y}-{\mathrm{y}}_i\right)}^2}-\sqrt{{\left(\mathrm{x}-{\mathrm{x}}_1\right)}^2+{\left(\mathrm{y}-{\mathrm{y}}_1\right)}^2}\\ {}=c\times \left(\mathrm{Tporpi}-\mathrm{Tporp}1\right)\end{array}} $$

In the formula, S_*i*1_ represents the distance difference between the mobile station, the base station *i* and the base station 1. We obtain a figure for the position of the mobile station using Formula (12).

### Taylor series expansion algorithm

We expand Formula (9) using the Taylor series expansion algorithm by removing two order and above components [6]. Formula (9) is converted to

10 $$ \upvarphi =\mathrm{h}-\mathrm{G}\updelta $$

In the above formula,

11 $$ \mathrm{h}=\left[\begin{array}{c}{S}_{2,1}-\left({S}_2-{S}_1\right)\\ {}{S}_{3,1}-\left({S}_3-{S}_1\right)\\ {}\vdots \\ {}{S}_{i,1}-\left({S}_i-{S}_1\right)\end{array}\right] $$

12 $$ \updelta =\left[\begin{array}{c}\varDelta x\\ {}\varDelta y\end{array}\right] $$

13 $$ \mathrm{G}=\left[\begin{array}{cc}\left[\Big({\mathrm{x}}_1-\mathrm{x}\right)/{\mathrm{S}}_1\left]-\left[\Big({\mathrm{x}}_2-\mathrm{x}\right)/{\mathrm{S}}_2\right]& \left[\Big({\mathrm{y}}_1-\mathrm{y}\right)/{\mathrm{S}}_1\left]-\left[\Big({\mathrm{y}}_2-\mathrm{y}\right)/{\mathrm{S}}_2\right]\\ {}\left[\Big({\mathrm{x}}_1-\mathrm{x}\right)/{\mathrm{S}}_1\left]-\left[\Big({\mathrm{x}}_3-\mathrm{x}\right)/{\mathrm{S}}_3\right]& \left[\Big({\mathrm{y}}_1-\mathrm{y}\right)/{\mathrm{S}}_1\left]-\left[\Big({\mathrm{y}}_3-\mathrm{y}\right)/{\mathrm{S}}_3\right]\\ {}\vdots & \vdots \\ {}\left[\Big({\mathrm{x}}_1-\mathrm{x}\right)/{\mathrm{S}}_1\left]-\left[\Big({\mathrm{x}}_{\mathrm{i}}-\mathrm{x}\right)/{\mathrm{S}}_{\mathrm{i}}\right]& \left[\Big({\mathrm{y}}_1-\mathrm{y}\right)/{\mathrm{S}}_1\left]-\left[\Big({\mathrm{y}}_{\mathrm{i}}-\mathrm{y}\right)/{\mathrm{S}}_{\mathrm{i}}\right]\end{array}\right] $$

Therefore, the least-square solution of the above formula is

14 $$ \delta =\left[\begin{array}{c}\Delta \mathrm{x}\\ {}\Delta \mathrm{y}\end{array}\right]=\left({\mathrm{G}}^{\mathrm{T}}{\mathrm{Q}}^{-1}\mathrm{G}\right)\ {\mathrm{G}}^{\mathrm{T}}{\mathrm{Q}}^{-1}\mathrm{h} $$

In this formula, Q is a covariance matrix of TDOA, and *δ* represents the revised value of this iteration. We get *x*^′^ = *x*_0_ + *Δx*, y^′^ = *y*_0_ + *Δy*, and then we repeat the above steps until the *δ* meets the requirements.

The initial value of the Taylor series expansion algorithm determines the complexity of the algorithm. When the initial value is not suitable, the computation time of the Taylor series expansion algorithm takes longer, the position accuracy will be greatly affected, and the algorithm cannot obtain the expected solution.

It is very important to select the most suitable initial value. Hence, this paper proposes a cooperative positioning algorithm. First, the Chan algorithm and the Fang algorithm are used to obtain the two groups of position coordinates. Second, the two groups of position coordinates are then weighted to obtain the initial value of the Taylor series expansion algorithm. The algorithm is described as follows:

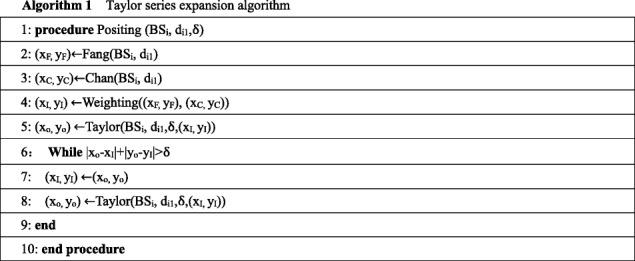



In this procedure, the (x_F_, y_F_) represents the result of the Fang algorithm, the (x_C_, y_C_) represents the result of the Chan algorithm, the (x_I,_ y_I_) represents the initial value, and the (x_o,_ y_o_) represents the positioning result.

### Simulation and analysis

To verify the performance of the algorithm, it is necessary to perform a simulation and analysis. In this paper, Matlab is used to simulate the algorithm. First, we need to determine the weight of both the Fang and Chan algorithms. We get a probability of position error of less than 20 cm, when the measurement error is set to 10, 20, 30, and 40 cm respectively. As shown in Table 1, we select the probability when the measurement error is 10 cm as the weight value.

**Table 1:** Probability of position error less than 20 cm

Measurement error (cm)	Fang algorithm	Chan algorithm
10	0.63	0.71
20	0.39	0.52
30	0.24	0.31
40	0.21	0.28

Next, we select the points on the y = 500 line as the actual coordinates of the target nodes, and assume the measurement error in the simulation process is 10 cm. The reason why the points on the y = 500 line are selected as the actual coordinates of the target nodes, is that this line can be clearly observed. To obtain the comparison diagram between the estimated position and the actual position under the cooperative position algorithm, we carried out the simulation 1000 times.

**Fig. 4:**
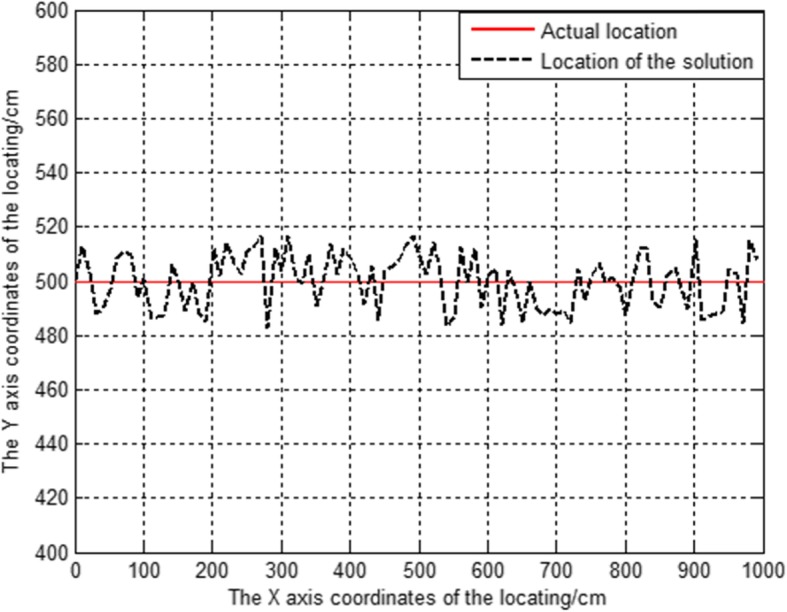
Position curve of target node

As shown in Fig. 4, we can see that the position algorithm in this paper can obtain the correct position result under these simulation conditions. The position result is floating up and down on the real position, and the position error is within 15 cm.

We ran the Fang algorithm and the cooperative algorithm 5000 times each to obtain a scatter diagram of the position result.

**Fig. 5:**
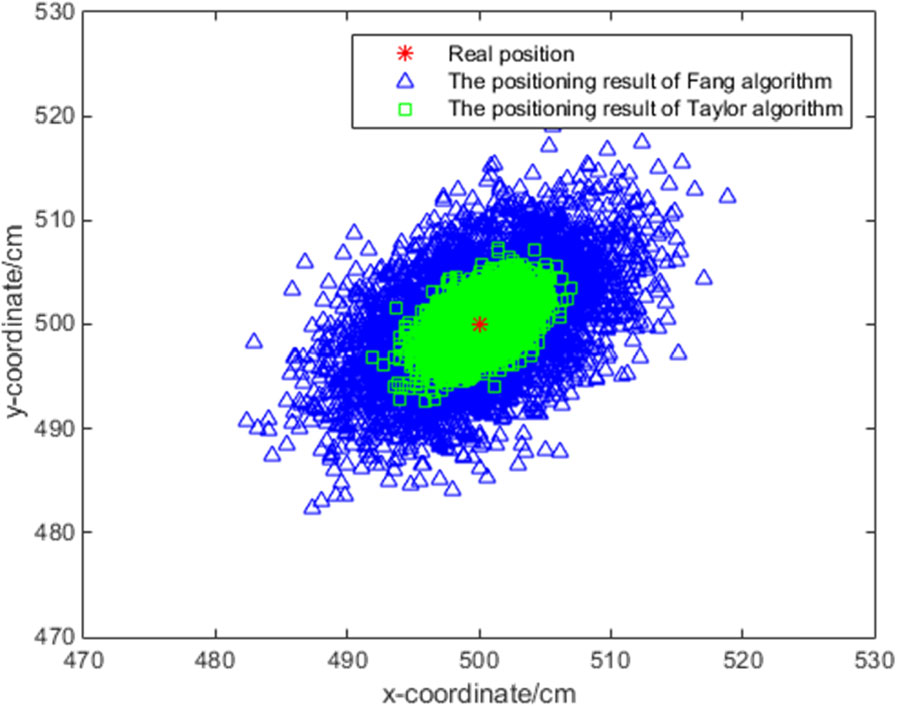
Scatter diagram of position result

Figure 5 shows that the positioning accuracy of the algorithm proposed in this research is higher than the accuracy of the Fang algorithm.

We set the actual position coordinates of the mobile station as (x_*r*_, y_*r*_), and then set the position result coordinates which was simulated and calculated by the position algorithm as (x, y). The formula for error calculation is as follows:

15 $$ \updelta =\sqrt{{\left({\mathrm{x}}_r-\mathrm{x}\right)}^2+{\left({\mathrm{y}}_r-\mathrm{y}\right)}^2} $$

In this formula, the δ represents the position error used to establish whether the position effect is positive or negative.

Then, we assume the real coordinates of the mobile station is MS (500, 500), and the coordinates of the three base stations are BS1 (0, 0), BS2 (1000, 0) and BS3 (0, 1000) respectively. The mobile station is surrounded by the base stations. The measurement error in this simulation process is still 10 cm, and the simulation was conducted 10,000 times to obtain the error curve.

**Fig. 6:**
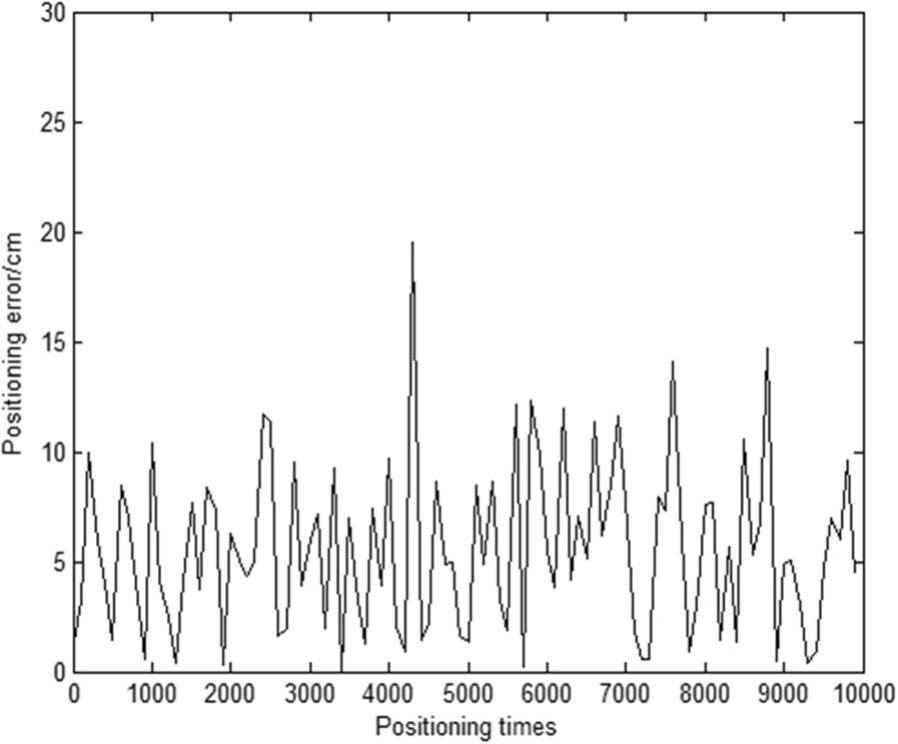
Position error curve

As Fig. 6 shows, under these simulation conditions, the position results obtained by this algorithm are more accurate, with position errors predominantly within 15 cm.

### Construction of the positioning system

**Fig. 7:**
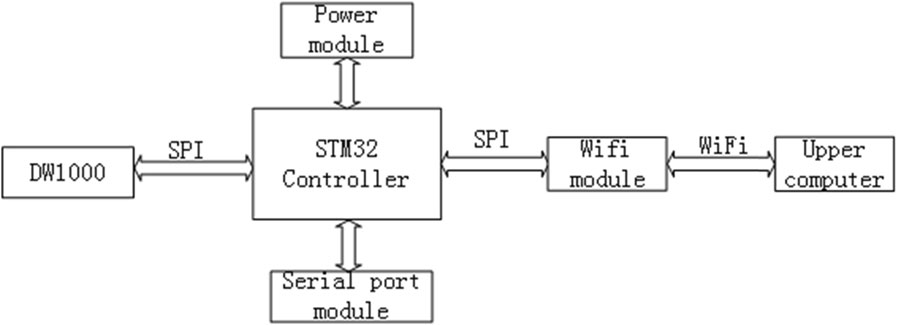
Block diagram of positioning system

The overall design block diagram of the UWB position system is shown in Fig. 7. The hardware circuit of the UWB positioning system is described as follows.

1. STM32 main control system. We select the STM32F103C8T6 microcontroller as the main controller of the UWB system. The core of this chip adopts ARM 32 Cortex^TM-M3 kernel architecture. With embedded memory consisting of 64 KB FLASH and 20 KB SRAM, which supports high-speed access, this technology also includes three low power modes, such as sleep, standby, and shutdown. The supply voltage of the STM32F103C8T6 microcontroller is 3.3 V, but the supply voltage of the proposed system is 5 V. Therefore, we use a LM1117–3.3 voltage regulator to convert the 5 V into 3.3 V, which provides a stable working voltage for the whole control chip.
2. DW1000 module. The DW1000 module is a wireless transceiver module that meets the IEEE802.15.4–2011 UWB standard. It is based on the DW1000 chip from Decawave, which can achieve position precision at centimeter level. It supports a data transmission rate up to 6.8 Mb/s and is especially suitable for wireless sensor network applications. Bidirectional ranging and positioning is also sustained.
3. Wi-Fi communication and power circuit. The wireless transmission module used in this work is nRF905, which is a single chip transceiver. The transceiver works in the ISM band of 433/868/915 MHz. It includes a fully integrated frequency modulator, a demodulator receiver, a power amplifier, a crystal oscillator.
4. Serial port conversion chips. To set and update the stations via a PC, we equip the base station and the move station with serial port conversion chips. In this research, a MAX3232 chip is used as the serial port conversion chip.

The performance of the proposed system was also verified in an indoor environment. We placed a mobile station in a room, surrounded by three base stations located in three corners of the room. We then conducted five tests and recorded the results as follows.

As shown in Table 2, we found that the algorithm can obtain positioning results, with an accuracy reach of approximately 15 cm.

**Table 2:** Test results of the system

Actual position	Measured value	Positioning error (m)
(1.0, 1.0)	(1.18, 1.15)	0.23
(3.0, 3.5)	(2.87, 3.46)	0.13
(5.0, 6.5)	(5.17, 6.38)	0.20
(7.0, 7.5)	(7.15, 7.45)	0.15
(9.0, 9.0)	(9.08, 9.24)	0.25

## Conclusions

To solve the defects of current indoor positioning systems, this paper proposes an indoor positioning system based on UWB. The system uses UWB as a means of communication and takes full advantage of the positive characteristics of UWB technology. The positioning system uses a cooperative positioning algorithm. After analyzing the results of our simulation, we can confirm that the algorithm can be characterized by low complexity and high position accuracy. This system has the potential to be utilized across a wide range of applications due to its high precision positioning in more complex indoor environments.

## Data Availability

Data sharing is not applicable to this article as no datasets were generated or analysed during the current study.

## Abbreviations

AOA: Angle of Arrival
FCC: Federal Communications Commission
RSSI: Received Signal Strength Indication
TDOA: Time Difference of Arrival
TOA: Time of Arrival
UWB: Ultra-wide bandwidth
